# Co-infection tuberculose-VIH compliquée d’une sur infection nosocomiale à *Klebsiella pneumoniae*: à propos de 4 observations dans un Service de Maladies Infectieuses au Mali

**DOI:** 10.11604/pamj.2020.37.141.22716

**Published:** 2020-10-08

**Authors:** Hermine Meli, Yacouba Cissoko, Issa Konaté, Mariam Soumaré, Assetou Fofana, Jean Paul Dembélé, Mikaila Kaboré, Mohamed Aly Cissé, Abdoulaye Zaré, Sounkalo Dao

**Affiliations:** 1 Service des Maladies Infectieuses, Centre Hospitalier Universitaire du Point G Bamako, Bamako, Mali,; 2 Faculté de Médecine et d´Odontostomatologie Bamako, Bamako, Mali,; 3 Centre de Recherche et de Formation sur la Tuberculose et le VIH, Bamako, Mali

**Keywords:** Tuberculose, *Klebsiella pneumoniae*, Centre Hospitalier Universitaire du Point G, Tuberculosis, Klebsiella pneumoniae, Point G University Hospital

## Abstract

Les infections nosocomiales constituent un problème majeur de santé publique dans le monde. La co-infection VIH-Tuberculose pulmonaire augmente la fréquence des infections nosocomiales, du fait de l´immunodépression et des hospitalisations itératives. Nous présentons quatre (04) patients âgés de 28, 36, 42 et 52 ans, co-infectés de VIH et tuberculose, (tuberculose multifocale chez 2, miliaire tuberculeuse, tuberculose bactériologiquement confirmée), tous ayant un taux CD4 < 100 cellules/mm^3^. Ils étaient à la phase intensive du traitement antituberculeux et sous antirétroviral (ARV). Ils avaient été admis au Service de Maladies Infectieuses du CHU du Point G pour toux productive, et/ou hyperthermie après des séjours antérieurs de plus de 48 heures en milieux hospitaliers. L´anamnèse avait révélé que l´un n´avait pas été observant au traitement antituberculeux du fait des effets indésirables classés mineurs. Il n´y avait pas eu d´amélioration clinique chez les 3 autres malgré une observance optimale aux différents traitements. L´examen cytobactériologique des expectorations et/ou du liquide de tubage gastrique, les hémocultures, prélèvements spécifiques avaient permis d´isoler Klebsiella pneumoniae multirésistante. Sous antibiothérapie spécifique, l´évolution clinique de ces patients avait été favorable. Les infections nosocomiales peuvent faire penser à tort à une mauvaise réponse thérapeutique lors d´un traitement antituberculeux. L´examen cytobactériologique des liquides biologiques doit être systématique chez les patients co-infectés de VIH-tuberculose. Notamment chez ceux ayant eu des séjours hospitaliers d´au moins 48 heures, et chez qui les signes pulmonaires et/ou la fièvre persistent en dépit d´une bonne observance aux traitements.

## Abstract (en)

Nosocomial infections are a real global public health problem. Pulmonary tuberculosis and HIV co-infection increases the incidence of nosocomial infections because of immunosuppression and iterative hospitalizations. We here report four cases of patients aged 28, 36, 42 and 52 years co-infected with HIV and tuberculosis (multifocal tuberculosis in 2 patients, miliary tuberculosis, bacteriologically confirmed tuberculosis), all with CD4 < 100 cells/mm^3^. During the intensive-phase, patients received antitubercular drugs and antiretroviral therapy (ART). They had been admitted to the Department of Infectious Diseases at the G-Point University Hospital with productive cough and/or hyperthermia following hospital stay greater than 48 hours. Patients’ history revealed that one patient had not been compliant with tuberculosis treatment because of adverse reactions classified as minor. There had been no clinical improvement in the three remaining patients despite their optimal compliance to different treatments. Cytobacteriological examination of sputum and/or testing of the feeding tubes, blood cultures and specific samples allowed to identify multidrug-resistant Klebsiella pneumoniae. The clinical course of these patients was favorable under specific antibiotic therapy. Nosocomial infections may be misinterpreted and associated with poor therapeutic response in patients receiving TB treatment. Cytobacteriological examination of the biological fluids should be systematic in patients co-infected with HIV and tuberculosis and hospitalized for at least 48 hours, in whom pulmonary signs and/or fever persist despite their good compliance to treatment.

## Introduction

Le VIH et la tuberculose constituent une association meurtrière. Cause majeure de mortalité chez les personnes vivant avec le VIH, la tuberculose est responsable de 13% de décès dans le monde [1]. En Afrique subsaharienne, le VIH est le principal déterminant de la hausse de l´incidence de la tuberculose observée au cours de ces 10 dernières années [1]. Le traitement de la co-infection VIH-tuberculose est identique à celui de la mono infection tuberculose. La guérison du patient dépend principalement de l´observance au traitement antituberculeux [2]. Cependant, le taux de rechute est parfois plus élevé, sans que l´on puisse toujours dire s´il s´agit d´une véritable rechute ou d´une réinfection [3]. Au cours du traitement, des agents infectieux responsables d´infections nosocomiales et/ou communautaires peuvent se greffer au tableau clinique initial aggravant ainsi la symptomatologie, et compromettant la guérison du patient. Nous rapportons quatre (04) cas de co-infections tuberculose-VIH type I. Ces patients étaient sous traitement antituberculeux catégorie 1, et thérapie antirétrovirale de première ligne (TDF/3TC/EFV) avec évolution clinique défavorable. L´un était non observant au traitement anti tuberculeux du fait d´effets indésirables classés mineurs, mais sous ARV bien conduit. Les 3 autres avaient une adhérence optimale aux 2 traitements. Après renforcement de l´observance des traitements et investigations paracliniques, *Klebsiella pneumoniae* multirésistante avait été isolé dans les prélèvements biologiques.

## Patient et observation

**Observation 1:** en juin 2018, un patient de 52 ans, infecté par le VIH type 1 est reçu au service des maladies infectieuses du point G. Sous Tenofovir 300mg /Lamivudine 300mg /Efavirenz 600mg en combinaison fixe, initié 3 semaines après le dépistage du VIH1 dans une structure hospitalière de la place. Son taux de CD4 à l´initiation était 18 cellules/mm^3^. Il avait été réadmis en hospitalisation après un précédent séjour de 21 jours au cours duquel il avait été diagnostiqué d´une tuberculose multifocale (pulmonaire, péritonéale) et soumis au traitement antituberculeux catégorie 1. Ce diagnostic avait été posé devant les arguments épidémiologiques, cliniques et paracliniques. Réadmis pour toux productive avec expectoration purulente et hyperthermie apparues 7 jours après son exéat, l´anamnèse avait retrouvé une mauvaise observance au traitement anti tuberculeux du fait du nombre important de comprimés et des effets indésirables à type de vertiges. Notre prise en charge a consisté tout d´abord en un renforcement de l´observance des traitements antirétroviral, antituberculeux et la recherche étiologique de la toux et des vertiges. Le contrôle des bacilles acido-alcoolo-résistants (BAAR) à la microscopie et le GenXpert des expectorations ont été négative à M1, M2. L´examen cytobactériologique des expectorations réalisé devant la persistance de la fièvre avait permis de mettre en évidence, *Klebsiella pneumoniae* résistant à toutes les bêtalactamines, sensible seulement à la Kanamycine, Amikacine, Chloramphénicol, colistine et Fosfomycine. L´évolution était déjà favorable à J3 de traitement sous Amikacine injectable, 15mg par Kg/jour et chloramphénicol injectable 50mg/kg/ jour.

**Observation 2:** en septembre 2018, est admis un homme de 42 ans, immunodéprimé au VIH type 1 dépisté depuis 3 ans, et naïf de traitement anti rétroviral avec un taux de CD4 initial à 38 cellules/mm^3^. Il avait été tabagique à 18 paquets années, et avait été sevré 02 mois avant son admission au service de maladies infectieuses. Hospitalisé pour fièvre persistante, asthénie et toux productive chronique, il était traité pour miliaire tuberculeuse non bacillifère. L´initiation au traitement antirétroviral suivant le schéma thérapeutique TDF300mg/3TC 300mg/EFV 600mg) s´était faite à S2 de traitement anti tuberculeux (RHEZ). Bien qu´observant aux traitements antirétroviral et antituberculeux, il a présenté à S10, une toux productive à expectorations purulentes devant laquelle la recherche de BAAR/GenXpert étaient négatives. L´examen cytobactériologique des expectorations demandé avait isolé *Klebsiella pneumoniae* sensible seulement à la Cefoxitine, Gentamycine, Colistine et Chloramphénicol. Sous Gentamycine à 3mg/kg/jour et chloramphénicol 500mg comprimés 1 comprimé toutes les 12 H, à J3 l´évolution clinique était déjà favorable.

**Observation 3:** patient de 36 ans, infecté par le VIH type 1, admis au service en novembre 2018 pour céphalées rebelles aux antalgiques usuels et fièvre à 40°C. Il avait été dépisté et initié sous le schéma TDF 300mg/3TC 300mg/EFV 600mg, 7 mois avant son hospitalisation au Service des Maladies Infectieuses. Son taux de CD4 à l´initiation était 84 cellules/mm^3^. L´examen physique à l´entrée avait retrouvé des signes d´irritations méningés et un syndrome inflammatoire à réponse systémique. La cytologie du liquide cerébro spinal d´aspect clair était lymphocytaire à 99%, avec une protéinorachie élevée 1,9g/l, hypo glycorachie et coloration de Ziehl Nielsen négative. La recherche active de BAAR était positive dans le liquide de tubage gastrique. Traité pour tuberculose multifocale (tuberculose pulmonaire et neuroméningée probable) à base de RHEZ, son exéat avait été autorisé devant la bonne évolution clinique à J18 de traitement antituberculeux. L´évolution à J 28 de traitement antituberculeux fut marquée par la réapparition d´une fièvre isolée oscillante entre 39 et 40°C. A noter que l´observance aux traitements ARV, et anti tuberculeux était optimale. La recherche étiologique de la réapparition de la fièvre s´était imposée à travers la microscopie/ GeneXpert, examen cytobactériologique (ECB) du liquide de tubage gastrique et les hémocultures. La microscopie et GeneXpert étaient négatives, et l´ECB avait permis d´isoler *Klebsiella pneumoniae* résistante à toutes les céphalosporines, sensible à la Netilmicine, Gentamycine, et Imipenème. Sous Gentamycine à 3mg/kg/jour en IVDL, et imipenème 1g/08h en intraveineuse directe, l´apyrexie était déjà observée à J2 de traitement.

**Observation 4:** le 16/09/2019 une dame de 28 ans infectée par le VIH1, naïve de traitement ARV est admise pour toux productive chronique en contexte fébrile. Son taux de CD4 était 75 cellules/mm^3^. La recherche de BAAR dans les expectorations était positive, *Mycobacterium tuberculosis* détectée au GenXpert avec sensibilité à la rifampicine. Elle avait été initiée au traitement ARV suivant le schéma TDF/3TC/EFV à J12 de traitement antituberculeux. Cependant, la fièvre a persisté (39 - 40°C) à J18 de traitement anti tuberculeux bien conduit. L´évolution marquée aussi par l´apparition d´une pollakiurie, des leucorrhées jaunâtres et abondantes. L´examen bactériologique du prélèvement vaginal avait mis en évidence *E. coli* sensible à l´amikacine, résistant aux quinolones et aux Bêtalactamines (excepté la Cefoxitine à sensibilité intermédiaire). Les carbapénèmes n´avaient pas été testés. L´hémoculture en aérobiose avait permis d´isoler *Klebsiella pneumoniae* sensible seulement à l´amikacine. L´examen cytobactériologique des urines avait isolé de nombreux bacilles Gram négatif, quelques Cocci Gram positif avec culture stérile. La bi antibiothérapie administrée était l´Imipenème 1g (empirique) toutes les 08h et amikacine 1g (spécifique) dans 400cc de sérum glucosé 10% en intraveineuse lente. A J4 de ce traitement, il y´avait déjà une apyrexie et disparition des autres signes sus cités.

## Discussion

Les pneumopathies bactériennes sont une cause importante de morbidité et de mortalité chez les patients infectés par le VIH [4]. La tuberculose pulmonaire est systématiquement recherchée chez les patients infectés par le VIH, qu´ils présentent des signes pulmonaires ou non. Également chez les patients présentant une pneumopathie communautaire dans les pays à forte endémicité de tuberculose. Les présentations cliniques et radiologiques de ces deux entités sont souvent similaires [5]. Il n´en demeure pas moins qu´une infection nosocomiale non diagnostiquée puisse compliquer le tableau clinique et entraver l´efficacité du traitement antituberculeux chez les patients en hospitalisation, et plus encore, ceux infectés par le VIH. Dans notre série, les 4 patients avaient eu séjours de plus de 48 heures dans un établissement hospitalier. S´il est vrai que l´un n´avait pas été observant au traitement tuberculeux, les 3 autres, malgré une observance optimale aux traitements antirétroviral et anti tuberculeux avaient eu une évolution clinique défavorable. La non-observance au traitement antituberculeux, et les conséquences graves qu´elles engendrent telles la rechute et la résistance bactérienne, représente le principal obstacle à une prise en charge correcte de la tuberculeuse, défi majeur dans les pays développés et en développement [6].

Chez le premier patient, l´examen cytobactériologique des expectorations avait été demandé en vue de rechercher une co-infection bactérienne bien que l´inobservance au traitement antituberculeux aurait clairement pu être évoquée comme l´une des causes évidentes de la persistance de la symptomatologie pulmonaire. *Klebsiella pneumoniae*, avait été isolée à l´examen cytobactériologique des expectorations des quatre patients et pouvait s´expliquer par des séjours dans des structures hospitalières. Afane *et al*. retrouvent *Klebsiella pneumoniae* chez les patients ayant séjourné en milieu pneumologique de l´hôpital Jamot indépendamment du statut sérologique au VIH [7]. *Klebsiella pneumoniae* est une entérobactérie communautaire, mais le plus souvent nosocomiale responsable des infections pulmonaires et urinaires. Hoza *et al*. retrouvent 9,7% des pneumonies non tuberculeuses chez les patients présentant une tuberculose pulmonaire en Tanzanie [5]. Gröschel *et al*. présentent une série de 02 cas de sepsis à *E. coli*, et de *Klebsiella pneumoniae* sécrétrices de B-lactamases chez des patients atteints de MDR-TB. Après instauration d´une antibiothérapie spécifique, l´évolution clinique a été favorable [8]. Cependant, la co-infection tuberculose pulmonaire et infections nosocomiales semble être sous diagnostiquée ou peu décrite chez les personnes infectées par le VIH.

Notre deuxième patient avait un antécédent de tabagisme, sevré deux mois avant son admission dans le service. Le tabac est un facteur de risque connu des infections pulmonaires à bacille Gram négatif. Sur le terrain d´immunodépression, ces infections sont généralement polymicrobiennes et peuvent être communautaires ou nosocomiales. Si l´infection à *K. pneumoniae* est habituellement retrouvée chez les malades diabétiques ou éthyliques [9], la présence de ces deux facteurs de risque n´a pas été relevé chez nos patients. La 3^e^ observation démontre la réapparition de la fièvre chez un patient co-infecté de tuberculose multifocale et VIH qui avait initialement une bonne évolution clinique. Les hémocultures réalisées sur milieux aérobie, anaérobie et fongiques étaient stériles. Il a fallu activement rechercher l´agent infectieux dans le liquide de tubage par l´ECB qui a permis d´isoler *K. pneumoniae* multirésistant. Au cours de la 4^e^ observation, nous avons décrit des infections nosocomiales à Gram négatif survenues sur un terrain de co-infection tuberculose-VIH, conférant une résistance aux Bêtalactamines et céphalosporines de 3^e^ génération. Une étude menée en Afrique du Sud démontre que la présence d´infections nosocomiales à Staphylocoques meti-résistants et entérobactéries sécrétrices de Béta lactamases chez les tuberculeux augmente le taux de mortalité [10]. Cependant dans notre série, nous n´avons déploré aucun décès bien que tous nos patients présentaient un état d´immunodépression sévère.

## Conclusion

Nous avons décrit quatre cas d´infections nosocomiales à *K. pneumoniae* chez les patients avec co-infection tuberculose-VIH, diagnostiqués devant la persistance des signes infectieux sous traitements anti rétroviral et antituberculeux. L´originalité de notre travail réside en la recherche étiologique des évènements cliniques survenant chez des patients sous traitements antituberculeux ayant eu un séjour récent en milieu hospitalier. Bien que peu documentée, l´association co-infection VIH-tuberculose et infection nosocomiale ne semble pas rare. Il convient de ne pas seulement penser à une mauvaise observance aux différents traitements lorsque l´évolution clinique est défavorable. L´examen cytobactériologique du liquide de tubage gastrique, les hémocultures, et autres prélèvements spécifiques doivent être systématiques chez les patients co-infectés de VIH et tuberculose. Surtout chez ceux ayant fait au moins une hospitalisation récente et dont il y´a réapparition/persistance d´un ou plusieurs signes pulmonaires ou infectieux, alors que la recherche de BAAR de contrôle est déjà négative.
